# Endoglin Wild Type and Variants Associated With Hereditary Hemorrhagic Telangiectasia Type 1 Undergo Distinct Cellular Degradation Pathways

**DOI:** 10.3389/fmolb.2022.828199

**Published:** 2022-02-25

**Authors:** Nesrin Gariballa, Praseetha Kizhakkedath, Nadia Akawi, Anne John, Bassam R. Ali

**Affiliations:** ^1^ Department of Genetics and Genomics, College of Medicine and Health Sciences, United Arab Emirates University, Al-Ain, United Arab Emirates; ^2^ Zayed Center for Health Sciences, United Arab Emirates University, Al-Ain, United Arab Emirates

**Keywords:** endoglin, hereditary hemorrhagic telangiectasia type 1, HHT1, HRD1 E3 ubiquitin ligase, ERAD, CD105

## Abstract

Endoglin, also known as cluster of differentiation 105 (CD105), is an auxiliary receptor in the TGFβ signaling pathway. It is predominantly expressed in endothelial cells as a component of the heterotetrameric receptor dimers comprising type I, type II receptors and the binding ligands. Mutations in the gene encoding Endoglin (*ENG*) have been associated with hereditary hemorrhagic telangiectasia type 1 (HHT1), an autosomal dominant inherited disease that is generally characterized by vascular malformation. Secretory and many endomembrane proteins synthesized in the Endoplasmic reticulum (ER) are subjected to stringent quality control mechanisms to ensure that only properly folded and assembled proteins are trafficked forward through the secretory pathway to their sites of action. We have previously demonstrated that some Endoglin variants causing HHT1 are trapped in the ER and fail to traffic to their normal localization in plasma membrane, which suggested the possible involvement of ER associated protein degradation (ERAD) in their molecular pathology. In this study, we have investigated, for the first time, the degradation routes of Endoglin wild type and two mutant variants, P165L and V105D, and previously shown to be retained in the ER. Stably transfected HEK293 cells were treated with proteasomal and lysosomal inhibitors in order to elucidate the exact molecular mechanisms underlying the loss of function phenotype associated with these variants. Our results have shown that wild type Endoglin has a relatively short half-life of less than 2 hours and degrades through both the lysosomal and proteasomal pathways, whereas the two mutant disease-causing variants show high stability and predominantly degrades through the proteasomal pathway. Furthermore, we have demonstrated that Endoglin variants P165L and V105D are significantly accumulated in HEK293 cells deficient in HRD1 E3 ubiquitin ligase; a major ERAD component. These results implicate the ERAD mechanism in the pathology of HHT1 caused by the two variants. It is expected that these results will pave the way for more in-depth research studies that could provide new windows for future therapeutic interventions.

## Introduction

Hereditary hemorrhagic telangiectasia type 1 (HHT1; OMIM 187300), also known as Rendu-Osler-Weber syndrome, is an autosomal dominant inherited disease that is generally characterized by vascular malformation which can range from small cutaneous and mucous membrane telangiectases to large arteriovenous malformation (AVM) in the lungs, liver, brain, and gastrointestinal tracts (McAllister et al., 1994; Richards-Yutz et al., 2010). The disease affects 1 in 10,000 individuals, however the age of onset of the disease and phenotype penetrance may vary considerably amongst affected individuals (McDonald et al., 2015). By the age of 21 the majority of patients develop recurrent nasal bleeds (epistaxis), and by late adulthood telangiectases of the lips, hands and face become apparent in nearly all affected individuals. AVMs usually manifest as congenital lesions that vary significantly in terms of lesion sites, numbers, and severity of symptoms (McDonald, 2011). On the other hand, large AVMs often account for serious consequences such as stroke and fatal hemorrhages that can lead to death (Karabegovic et al., 2004). HHT1 has been associated with mutations in the transforming growth factor Beta (TGFβ) co-receptor Endoglin (also termed as cluster of differentiation 105, CD105) that is encoded by *ENG* (McAllister et al., 1994). Haploinsufficiency due to loss of function is widely accepted as the underlying functional mechanism for HHT1 (Galaris et al., 2021).

TGFβ signaling pathway plays a key role in diverse sets of cellular signaling activities during the early embryogenic developmental stages and also throughout adulthood (Hata and Chen 2016; Kashima and Hata 2018). The TGFβ signaling pathway regulates cell growth, differentiation, apoptosis, and immunological responses through a complex signaling systems with multiple components that control gene expression in a context dependent manner (de Caestecker 2004; Kashima and Hata 2018). The TGFβ signaling cascade is initiated with the ligand binding to the serine/threonine type II receptor dimer which recruits and phosphorylate a type I receptor forming a heterotetrameric complex (Shi and Massagué 2003). In addition to Endoglin, some other TGFβ signaling pathway components have been implicated in several single gene disorders involving vascular malformations (Gariballa and Ali, 2020).

**FIGURE 1:**
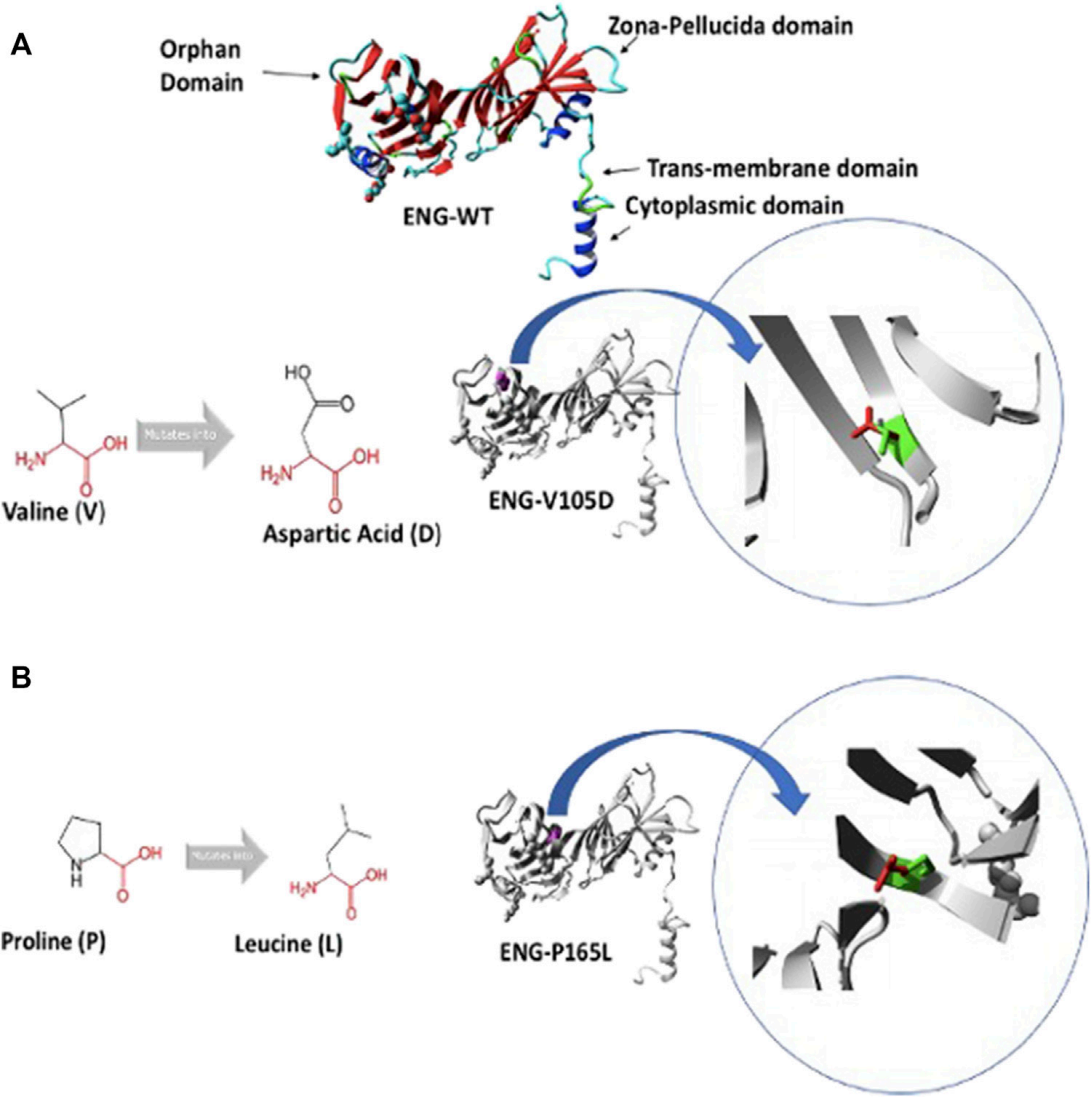
Predicted structure of Endoglin WT and variants V105D and P165L. **(A)** shows 3D ribbon structure of Endoglin WT built by HOPE protein modelling program based on a homologous structure. It shows mutation of amino acid variants Valine (V) to Aspartic acid (D) (Both in black). Focus on mutation site: The protein is coloured grey, the side chains of both the wild type “V” and the mutant residue “D” are shown and coloured green and red respectively.**(B)** mutation of amino acid variants Proline (P) to Leucine (L) (Both in black). Focus on mutation site: The protein is coloured grey, the side chains of both the wild type “P” and the mutant residue “L” are shown and coloured green and red respectively.

The Endoglin co-receptor has no intrinsic enzymatic activity, however it enhances and stabilizes the binding of ligands to the heterotetrameric receptors dimer (Kim et al, 2019). The signal is propagated to the nucleus through phosphorylation of selective sets of the transcription factors; SMADs, that can enter the nucleus and regulate gene expression (Groppe et al., 2008; Massagué 2008). Endoglin is a single membrane spanning receptor with a molecular weight of 90–95 kDa forming a homodimer that is stabilized by multiple disulphide bridges (Lux et al., 2000). The homodimer consists of two extracellular domains: N- terminal orphan domain (OR), C-terminal zona pellucida (ZP), a transmembrane domain and a short cytoplasmic domain (Figure 1). Excision of the extracellular domain gives rise to a soluble shorter isoform of Endoglin (Endoglin ^S^). The extracellular domains contain ligand binding sites as well as attachment sites for *N* and *O* glycosylation (Meurer and Weiskirchen 2020). X-ray crystallography studies have demonstrated the interaction of the ligand with hydrophobic (OR) domain, whereas protein homodimerization occurred through cystine bridges in the ZP domain (Saito et al., 2017). Endoglin is predominantly expressed in endothelial cells (EC) as a component of the receptor complex comprising type I receptor (ALK1) or ALK5, and type II receptor binding ligands such as bone morphogenetic protein 9 and 10 (BMP9 and BMP10) and transforming growth factor-β1 (TGF-β1) (Ollauri-Ibáñez, López-Novoa, 2017; Pericacho 2017). This signaling cascade leads to the activation of SMAD 1/5/8 transcription factors that enter the nucleus and upregulate genes that promote endothelial cells activation and control the whole mechanism of vasculogenesis (Guerrero-Esteo et al., 2002; Castonguay et al., 2011). Mutations in Activin receptor-like kinase gene (*ACVRL1*) encoding the type I receptor ALK1, a major partner in the TGFβ signaling pathway, has been associated with hereditary hemorrhagic telangiectasia type 2 (HHT2, OMIM 600376) (Johnson et al., 1996). Mutations in *ENG* and *ACVRL1* lead to similar phenotype and account for 85% of hereditary hemorrhagic telangiectasias (Gallione et al., 2004). The remaining cases are attributed to mutations in *SMAD4*, *GDF2* (encoding BMP9), and other yet unknown genes (Shovlin et al., 2020). Recently progress has been made in conventional therapies that targets the angiogenic molecular pathway using inhibitors of vascular endothelial cells factors (VECF) such as bevacizumab (anti-VEGF antibody) (Buscarini et al., 2019). Nonetheless, the molecular mechanisms underlying this life-threatening disease and the loss-of-function traits associated with the disease phenotype remains to be fully elucidated. Understanding the molecular mechanisms by which these mutant proteins lose their function could open up new windows for therapeutic interventions that potentiate protein functional rescue. We have previously demonstrated that defective trafficking of mutant Endoglin variants play a major role in the disease pathology (Ali et al., 2011). Sub-cellular localization of wild type Endoglin and 28 disease-causing variants have been investigated using confocal microscopy and the results have shown that more than 50% of the mutant variants are retained in the ER and failed to traffic to their normal localization on the plasma membrane (Ali et al., 2011).

TGFβ receptors including Endoglin are classical examples of secretory membrane proteins that undergo a series of quality control checks and posttranslational modifications in the ER and along the secretory pathway. The ER has adapted a highly conserved quality control mechanisms to ensure that only properly folded and assembled proteins can be dispatched to their functional sites (Sun and Brodsky 2019). In addition, the unfolded protein response (UPR) is triggered by unresolved mutant proteins that accumulate in the ER lumen and form aggregates that disrupt ER homeostasis. The three arms of the UPR mechanism, including PERK (protein kinase RNA-like endoplasmic reticulum kinase), Inositol-requiring kinase 1(IRE I) and activating transcription factor 6 (ATF6), trigger the expression of molecular chaperones that assist protein folding, and relief ER stress (Christianson and Ye 2014; Hwang and Qi 2018). Furthermore, the UPR mechanism aim to attenuate the translation mechanism in order to reduce the work load on the ER until normal cellular functions are restored (Preston and Brodsky 2017). Terminally misfolded proteins that fail to reach their proper conformation after cycles of encounters with activated ER molecular chaperones, will be targeted to the ER associated protein degradation (ERAD) pathway (Preston and Brodsky 2017). Misfolded proteins are recognized as ERAD substrates by resident ER molecular chaperones (EDEM1, OS9, and XTP3-B) that all work in concert with other co-chaperones to facilitate retrotranslocation of terminally misfolded proteins through the HRD-1/SEL-1L translocon channel to be degraded in the cytosol by the ubiquitin/proteasomal system (reviewed in (Hwang and Qi 2018; Gariballa and Ali 2020).

In this study, we demonstrate that the degradation pathways of wild type (WT) Endoglin is distinct from some disease-causing ER-retained mutant variants. Our data shows that WT Endoglin is degraded relatively quickly through both proteasomal and lysosomal pathways, whereas the mutant variants P165L and V105D, trapped in the ER, have a much longer half-lives, and get degraded predominantly through the proteasomal degradation pathways. Importantly, by means of CRISPR-Cas9 gene editing technique, we confirm the important role of HRD-1 E3 ubiquitin ligase in the retro-translocation of the mutant Endoglin variants P165L and V105D from the ER to the cytosol where they undergo proteasomal degradation. These findings further implicate ERAD in the pathogenesis of HHT1 disease and possibly open up new windows for therapeutic interventions.

## Materials and Methods

### Generation of Stably Transfected Cell Lines

Human Embryonic Kidney 293 (HEK293) cells were transiently transfected with pcDNA3.0 expression vector harboring the HA-tagged WT Endoglin or the missense mutant variants P165L and V105D designed through site directed mutagenesis described previously (Ali et al., 2011). However, the V105D variant has been incorrectly referred to in Ali et al. (2011) as V125D. pcDNA3.0 vector carries Neomycin resistance gene as a selection marker. For selection of stably transfected cells, 48 h after transfection, cells were grown in culture medium supplemented with 700 μg/ml G418 sulphate. Monoclonal cell lines were generated by limiting dilution in 96-well plates. Single cell clones generated were validated for stable transgene expression by immunoblotting and immunostaining against the HA tag. Stable cell lines carrying an empty pcDNA3.0 vector were also generated as negative control.

### Cell Culture, Transfection, and Treatments

HEK293 cells were cultured in Dulbecco’s modified Eagle’s medium (Invitrogen) supplemented with 10% FBS (Invitrogen), penicillin (10 U/ml) and streptomycin (100 μg/ml) at 37°C with 5% CO_2_. For transfection experiments, cells were grown in 6-well tissue culture plates and transfected with 1 µg plasmid DNA using FuGENE HD transfection reagent. Co-transfection with 0.5 µg plasmid vector harboring red fluorescence protein (RFP) (Thermo Fisher Scientific) was carried out as a transfection efficiency control for transient transfection of plasmid constructs carrying WT Endoglin and mutant variants.

For cycloheximide chase assays, stably transfected HEK293 cells were treated with cycloheximide (100 μg/ml) and harvested at specific time points (0, 2, 4, 8, 16, and 24 h). Cells were then lysed in RIPA buffer and kept at −80°C for Western blot experiments.

For treatments with proteasomal or lysosomal inhibitors, cells were grown in DMEM with a supplement of 10% FBS, penicillin (10 U/ml) and streptomycin (100 μg/ml) until they reach 60–70% confluency. Cells were then serum starved for 4–8 h followed by incubation in serum free medium containing the indicated proteasomal inhibitors (MG132 at 10 µM and Epoximycin at 100 nM), ERAD inhibitors (Eeyarestatin I at 5 µM/and Kifunensine at 50 nM), and lysosomal inhibitors (Bafilomycin at 200 nM).

### Protein Extraction, Western Blotting Analysis, and Immunoprecipitation

After cells were harvested and pelleted, protein extraction was carried out using RIPA lysis reagent (Sigma) supplemented with protease inhibitors cocktail (Sigmafast protease inhibitor cocktail). Cell lysates were then quantified using Bicinchoninic Acid protein Assay (BCA kit, and Pierce), according to manufacturer’s protocol. Equal amounts of protein were mixed with Laemmli loading buffer, heated to 95°C for 5 min and then resolved on an SDS-PAGE at concentrations relevant to the protein sizes. However, Endoglin was found to aggregate at the top of the acrylamide gel at 95°C, therefore cell lysates were mixed with Laemmli buffer plus Dithiothreitol (DTT, 5 mM) and heated at 55°C for 10 min prior to loading into the gel. This was followed by blotting onto a PVDF page and then probed with the respective antibodies at an optimized dilution. Detection was performed using Enhanced Chemiluminescence Plus reagent (ECL plus, Pierce) and then it was visualized using Typhoon FLA 9500 Imager (GE Healthcare Biosciences). ImageJ software was used for densitometric quantification analysis of immunoblots generated.

For immunoprecipitation, stably transfected HEK293 cells were lysed in IP lysis buffer (Pierce Inc.) supplemented with protease inhibitors cocktail (Sigmafast protease inhibitor cocktail). After total protein extraction and quantification (as described above), equal amounts of cell lysates were incubated with anti-HA agarose beads (Pierce) for 2 h at 4°C. Beads containing the immunoprecipitated proteins were collected by centrifugation and washed three times with lysis buffer. Proteins were eluted from the beads by boiling in Laemmli sample buffer to be used for western blotting.

### Endoglycosidase H Sensitivity and Resistance Assay

Immunoprecipitated proteins were denatured in 1× glycoprotein denaturation buffer (0.5% SDS and 1% β-mercaptoethanol) for 5 min at 100°C. The denatured proteins were incubated for 4 h at 37°C in the presence or absence of 10 U of endoglycosidase H (Endo H; Sigma-Aldrich). The samples were then resolved on 8% SDS/PAGE gels and analyzed by western blotting.

### Triton X-100 Solubility Assay

The assay was carried out as in (Houck et al., 2014). In short, harvested cells were lysed in TBS-Triton (50 mM Tris-Cl, 150 mM NaCl, 1% Triton X-100, and pH 7.6) and separated into soluble fraction (supernatant) and aggregated fraction (pellet) via 20,000 g centrifugation for 15 min at 4°C. 30 µg of cell lysates were added to SDS sample buffer with 1.25% β-mercaptoethanol and then resolved on an SDS-PAGE. GAPDH was used as a marker for the soluble fraction and Histone H3 was used as a marker for pelleted fraction.

### Generation of HEK293-HRD1 Knockout Cell Line Using CRISPR-Cas9 Gene Editing

KN2.0 non-homology mediated CRISPR kit (Origene inc.) was used for *HRD1* gene Knockout. The gene specific gRNA carrying the sequence (ACT​GTG​GTG​TAC​CTG​ACC​AA) leads Cas9 to cut the target genome, and the cutting site is repaired by the integration of predesigned linear donor containing a puromycin resistant gene for selection of cells that have the linear donor encoding the reporter gene (*GFP*) integrated. The gRNA vector plus the DNA donor were transfected into the cells using FuGENE HD transfection reagent. Scrambled gRNA was used as negative control. Cells were passaged 5 to 6 times before treatment with the pre-determined kill dose for puromycin (0.7 μg/ml) for 7 days for optimal selection of positive clones. Single cells were then seeded in a 96 well plated using serial dilution of cell culture. After 2 weeks of observation single cell wells were marked and grown on 6 well plates for DNA/RNA and protein extraction. Bi-allelic gene perturbation on the DNA level has been confirmed using Sanger sequencing and gene knockout on the protein level was validated using immunoblotting against HRD1.

### Immunocytochemistry

HEK293 cells, stably transfected with pcDNA3.0 expression vector harboring the HA-tagged WT Endoglin or the missense mutant variants P165L and V105D, were grown on coverslips. Cells were then fixed by methanol at −20°C for 4 min. Fixed cells were washed three times with PBS and incubated in (1% BSA in TBST) blocking solution for 30 min at room temperature. For immunofluorescence staining, the protocol was followed as in (Ali et al., 2011).

### Antibodies

Antibodies for Western blot analysis: Rabbit monoclonal anti-HA-tag (Cell Signaling Technology, at 1:1,000 dilution), Rabbit monoclonal anti-SYVN1 (Cell Signaling Technology, at 1:1,000 dilution), mouse monoclonal anti-GAPDH (Abcam, at 1:2,500 dilution) rabbit monoclonal Anti-HA (Cell Signaling, at 1:1,000 dilution), mouse monoclonal anti-RFP (Thermo Fisher Scientific, at 1:1,000 dilution), rabbit anti- Endoglin P3D1 (Santa Cruz Biotechnology, at 1:200 dilution), anti-Mouse IgG Peroxidase antibody (Sigma Aldrich, at 1:40,000 dilution), and anti-Rabbit IgG Peroxidase antibody (Sigma Aldrich, at 1:30,000 dilution).

Antibodies for immunofluorescence: mouse monoclonal anti-HA-tag (Cell signaling Technology, at 1: 200 dilution), rabbit polyclonal anti-calnexin (Santa Cruz Biotechnology, at 1 : 200 dilution), rabbit anti-Histone-H3 (Cell Signaling Technology, at 1:1,000 dilution), mouse monoclonal anti-HA (Cell Signaling Technology, at 1: 200 dilution), Alexa Fluor 568-goat anti-mouse IgG (Molecular Probes, at1:200 dilution), and Alexa Fluor 488-goat anti rabbit IgG (Molecular Probes, at 1:200dilution).

### In Silico Analysis of Endoglin Variants P165L and V105D

We have utilized HOPE software, which is a fully automated protein modelling program, for the prediction of functional, and structural effects of the two point mutations (P165L and V105D) in Endoglin (Venselaar et al., 2010).

### Statistical Analysis

Statistical analysis between each group and the control was conducted by one-sample unpaired t-test (GraphPad Prism software). For the time dependent cycloheximide chase assay comparison between mutants groups and wild type was conducted using two-way ANOVA and Dunnet’s multiple comparison test (GraphPad Prism software); (*) *p* ≤ 0.05; (**) *p* ≤ 0.01; (***) *p* ≤ 0.00. In all graphs, Error bars represent SEM from biological replicates indicated as the number (n) on the figure legends.

## Results

### Protein Modelling Reveals Functional and Structural Defects in ER Retained P165L and V105D Endoglin Variants

In an attempt to better understand the basis of misfolding and ER retention of the two Endoglin variants P165L and V105D, we used HOPE protein modelling which could shed light on the possible structural effects of the two point mutations in Endoglin and the possible consequences on their biological function (Figure 1). ENG-V105D variant carries a point mutation as a result of a substitution of amino acid Valine (V) to Aspartic Acid (D), both colored in black (Figure 1A). The new amino acid variant (D) is bigger and carries a negative charge compared to the wild variant which is smaller and has a neutral charge. WT residue (V) is also reported to be very conserved in that position within a stretch of residues annotated in Uniport as required for interaction with BMP9 ligand. HOPE has also predicted that substitution of Proline (P) to Leucine (L) in ENG-P165L variant to be damaging (Figure 1B). Both variants fall in the Orphan domain where two conserved disulfide bonds involving C30–C207 and C53–C182 are formed (Saito et al., 2017). The structural disorder caused by the P165L variants is predicted to disturb the cysteine bridge in this domain. The possible loss of cystine bonding probably account for both distortion of the 3D structure of the protein as well as exposure of buried hydrophobic residues and possible formation of new cystine bonding that may cause mutant protein aggregation.

### Proteasomal Inhibition by MG132 Causes Accumulation of Both WT Endoglin and Mutant Variants P165L and V105D and Partially Alters the Mobility of the Mutants on SDS-PAGE Gels

**FIGURE 2:**
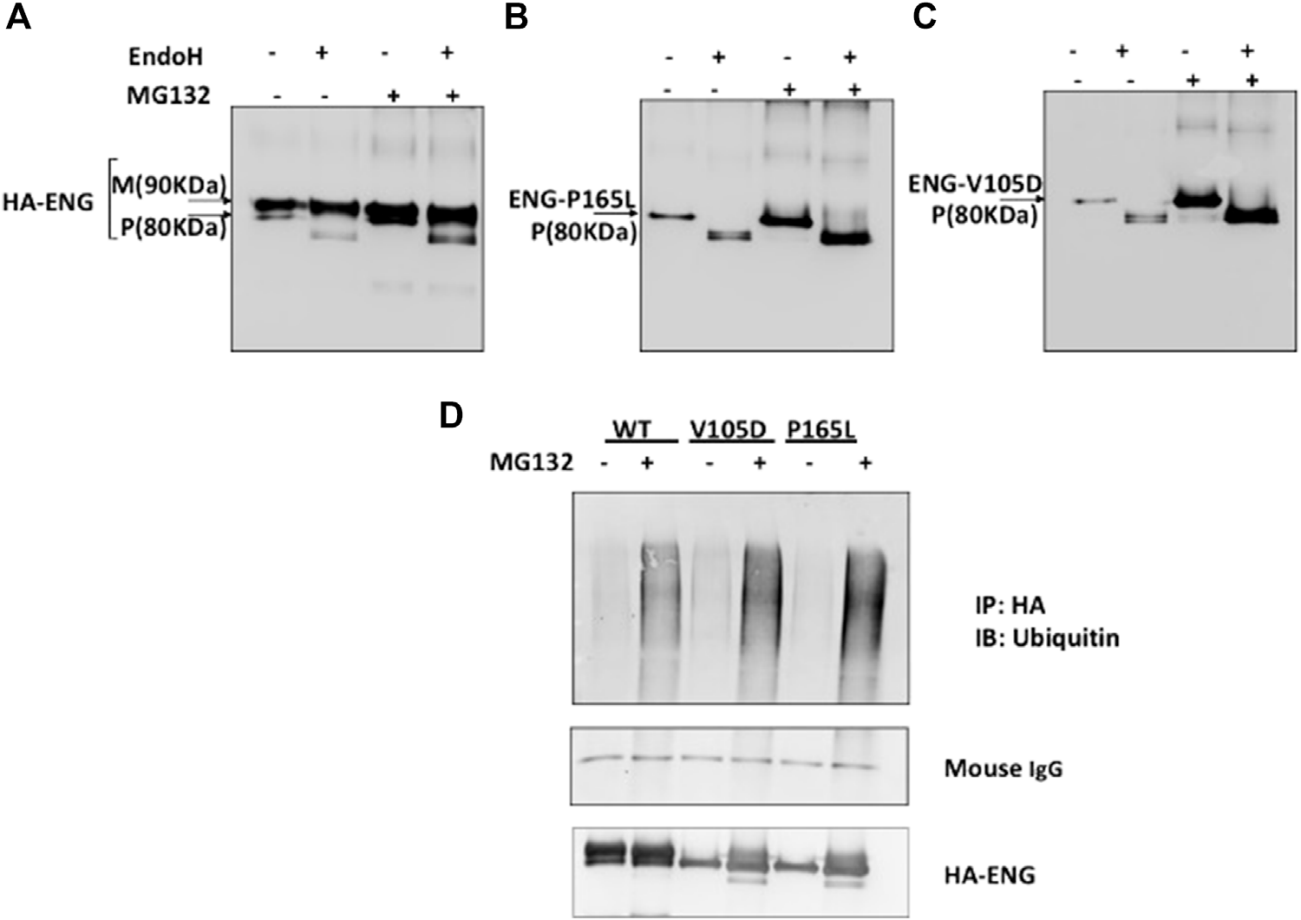
Proteasomal inhibition of WT Endoglin and mutant Variants P165L and V105D in transiently transfected HEK 293 cells. **(A–C)** Immunoblots of immunoprecipitated Endoglin species (WT, P165L, and V105D) digested with EndoH after treatment with proteasomal inhibitor (MG132) (10 μM). Endoglin mature band at 90 KDa is indicated by (M) and precursor band at 80 KDa is indicated by (P). **(D)** Immunoprecipitated Endoglin WT and mutant variants P165L and V105D were treated with 10 μM MG132 (+) or DMSO (−) for 16 h. The immunoprecipitates were immunoblotted with antibodies against Ubiquitin. Mouse IgG from IP beads are shown as loading control. The ubiquitin blots were stripped and re-probed against HA for confirmation.

We have demonstrated in a previous study that missense mutant variants L32R, C53R, V105D (referred to wrongly as V125D), P165L, I271N, W149R, D264N, and V311G transiently transfected into HeLa and HEK293 cells are trapped in the ER and failed to traffic to their indigenous cellular functional location at the plasma membrane (Ali et al., 2011). In order to investigate a possible role of the ERAD mechanism in the degradation of ER retained variants, HEK293 cell were transiently transfected with pcDNA3.0 vectors harboring HA tagged Endoglin WT and mutant variants P165L and V105D, then treated with the proteasomal inhibitor MG132 (10 μM). Immunoblotting analysis have shown an accumulation of both WT Endoglin and the mutant variants P165L and V105D after the treatment with MG132 (Figures 2A–C). This was followed by Endo H sensitivity and resistant assay in an attempt to investigate the glycosylation profiles of accumulated mutant variants P165L and V105D (Figures 2A–C). The concept underlying Endo H assay is that Endo H can cleave the carbohydrate moieties of ER localized N-glycoprotein, whereas post-ER proteins are protected by further modelling of the N- glycan in the Golgi complex that renders these glycoproteins resistant to Endo H cleavage. The Endo H assay for immunoprecipitated WT Endoglin and mutant variants P165L and V105D was carried out before and after the treatment of MG132. In both cases, WT Endoglin shows distinct two bands indicating the mature (M) and precursor (P) receptor protein at ∼80 and ∼90 KDa, respectively. Endo H treatment neatly cleaves the precursor band to lower molecular weight band (Figure 2A). On the other hand, Endo H digestion of Endoglin variants P165L and V105D after MG132 treatment seemed to result in alteration of Endoglin variants’ mobilities on the SDS-PAGE gels (Figures 2B,C). This was indicated by a cleavage of the precursor protein leaving behind a smear pattern at the position of the mature Endoglin band. This result which may indicate that proteasomal inhibition could be a potential target for mutant Endoglin variants’ rescue.

Protein degradation through the proteasomal pathway is signaled with the covalent attachment of a chain of ubiquitin molecules to protein substrate. In order to further consolidate our findings that mutant variants P165L and V105D are predominantly degraded through the proteasomal route, it was necessary to investigate their ubiquitination status prior to degradation. Immuno-precipitated Endoglin WT and mutants were immunoblotted against ubiquitin before and after treatment with proteasomal inhibitor MG132 (Figure 2D). Our results show accumulation of higher molecular weight ubiquitinated forms of both WT and mutant variants after MG132 treatment. However, mutant variants seem to be more heavily polyubiquitinated than WT Endoglin. These results further emphasize the role of the ubiquitin proteasomal pathway in the degradation of Endoglin variants associated with HHT1.

### Conformation Specific ENG-P3D1 Antibody Confirms Misfolding of Mutant Variants P165L and V105D in Stably Transfected HEK293 Cells

In this study, we have generated stably transfected HEK293 clonal cell lines harboring the WT Endoglin and two mutant variants P165L and V105D in order to elucidate the detailed degradation pathway of these two ER-retained variants and compare them with WT and hence shed light on the cellular mechanisms underlying their loss of function and degradation pathways. Confirmation of the generation of clones of these stably transfected cells has been validated through western blotting and immunofluorescence staining, presented in Supplementary Figures S1, S2, and S3, respectively.

**FIGURE 3:**
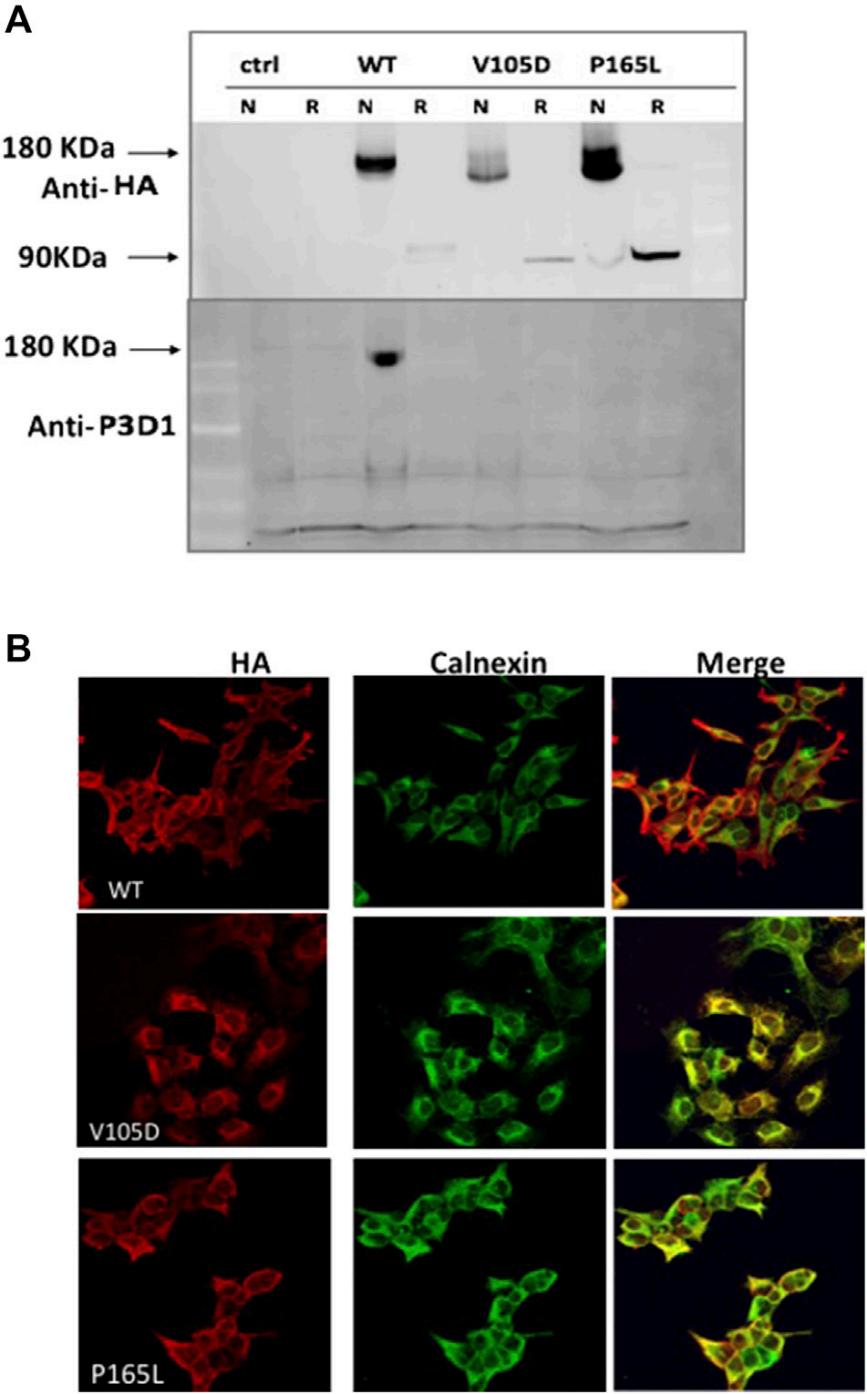
Conformation distortion and subcellular colocalization of mutant variants P165L and V105D. **(A)** Binding of the conformation specific P3D1 antibody to only the cell surface localized, non-reduced N-terminal epitope of ENG. Equal amount of cell lysate from stably transfected cells expressing the wild type or mutants were resolved under non-reducing (N) and reducing conditions and analyzed by both HA (top panel) and P3D1 (bottom panel) antibodies. **(B)** Immunofluorescence images showing colocalization of HA-tagged WT Endoglin and mutant variants P165L &V105D with ER marker (Calnexin) in stably transfected HEK-293 cells.

In order to investigate the tertiary conformation of Endoglin mutant variant P165L and V105D, cell lysates of stable clones generated were probed with the conformation specific antibody P3D1 (Figure 3A). The monoclonal antibody P3D1 recognize the N-terminal region of 204 amino acids encoded by exons 1–5 and has reduced affinity to Endoglin monomer (Pichuantes et al., 1997). We performed Western blot analysis under non-reducing conditions and reducing conditions. The cell lysates from stably transfected cell lines were denatured in sample buffer with or without a reducing agent and analyzed by western blotting. Our western blotting analysis revealed that the P3D1 antibody only detected the wildtype Endoglin under nonreducing conditions. The mutants were not recognized by the P3D1 antibody indicating that mutation interfere with native epitope conformation of the protein. Anti-HA antibodies were able to recognize both non-reduced and reduced forms of the mutant proteins, but the mobility of the dimers were distinct from that of the wild type dimer. In order to elucidate the retention of mutant variant P165L and V105D in the ER, cells were immunostained against Endoglin- HA tag (Red) and the ER marker (calnexin) (Green) (Figure 3B). Unlike WT Endoglin, mutant variants P165L and V105D colocalized predominantly with the ER marker (calnexin). Since no cell surface localized mutant Endoglin was detected in immunofluorescence images, mutant dimers recognized by anti HA antibody are most likely originated from the ER retained mutant forms. These results corroborate the inferences from the protein modelling data that the possible loss or gain of cysteine bonding in the orphan domain could lead to distortion of the 3D structure and possible mutant protein aggregation.

### The P165L and V105D Endoglin Variants are Highly Stable *In Vivo* Compared to the Wild Type Protein

**FIGURE 4:**
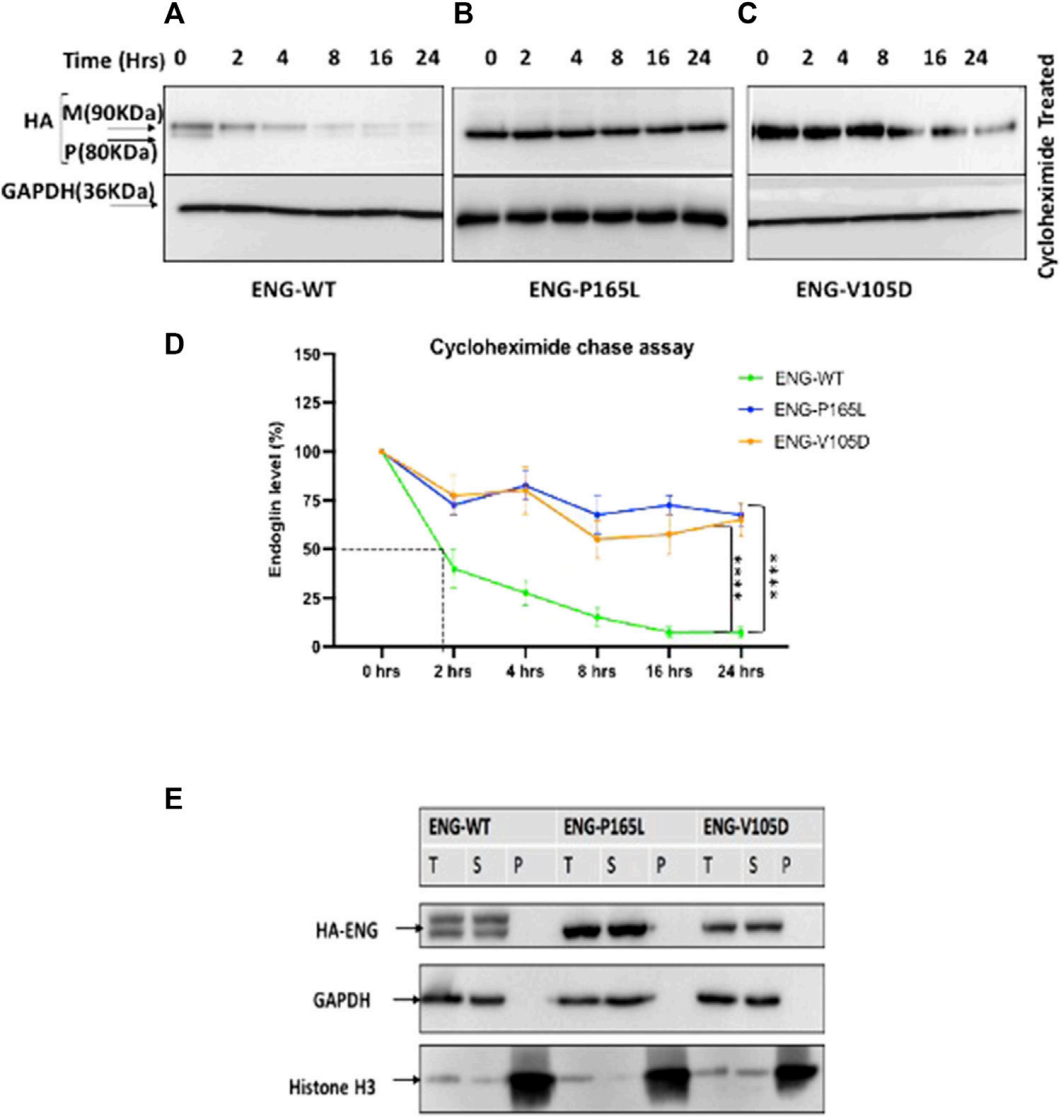
Cycloheximide (CHX) chase assay for stably transfected HEK293 Endoglin WT and the two mutant variants P165L and V105D. **(A–C)** HEK293 stably transfected with p. ENG-WT, p. P165L, and p. V105D were treated with Cycloheximide (100 μg/ml) for the time points 0, 2, 4, 8, 16, and 24 h. **(D)** Line graph for Endoglin Cycloheximide chase assay represents mean densities of WT Endoglin and mutants relative to untreated at 0 h, normalized with loading control (GAPDH). **(E)** Western blot analysis of Endoglin WT and mutant variants’ aggregation state. Harvested cells were lysed in 1%Triton lysis buffer (TBSt) followed by centrifugation at 20,000 g at 4°C. Supernatant (S), Pellet (P), and Total Lysate (T) were resolved in SDS page and then immunoblotted against HA-ENG. Histone H3 was used as a marker for P fraction and GAPDH for the T and S fractions. Error bars represent SEM from 4 independent experiments (*n* = 4). Statistical significance between two groups was assessed using repeated measures two-way ANOVA; (*) *p* ≤ 0.05; (**) *p* ≤ 0.01; (***) *p* ≤ 0.001.

To analyze the kinetic of Endoglin protein variants stability and degradation *in vivo*, cycloheximide chase experiments were carried out on HEK293 cells stably transfected with WT Endoglin and the two mutant variants P165L and V105D. Cycloheximide is a translation elongation inhibitor that blocks global protein synthesis and hence protein half-life can be determined. Stably transfected HEK293 cells were treated with cycloheximide and cell lysates were prepared at 0-, 2-, 4-, 8-, 16-, and 24-h intervals for Western blotting analysis (Figures 4A–C). In the immunoblots, drastic decline in protein ENG-WT level was observed over the chase experiment (Figure 4A). On the other hand, P165L and V105D variants remained relatively stable (Figures 4B,C). The line graph, depicting the densitometric analysis of the immunoblots of cycloheximide chase assays (*n* = 4), illustrates that WT Endoglin has a relatively short half-life of less than 2 hours (Figure 4D). A significant difference was detected between WT-ENG and both variants P165L and V105D over the course of the chase period (Figure 4D). Both mutant variants retained around 70% of their initial protein band intensity until the end of the 24-h chase period. However, our Triton X-100 solubility assay revealed that both Endoglin WT and the two mutant variants P165L and V105D are soluble in the non-ionic detergent (Triton X-100) and hence are not likely to have form detergent insoluble aggregates (Figure 4E). This was evidenced by the lack of any traces of Endoglin in the pelleted fraction of lysate that is likely to comprise the detergent insoluble protein aggregates.

### The P165L and V105D Variants Show Distinct Degradation Pathway Compared to Wild Type

**FIGURE 5:**
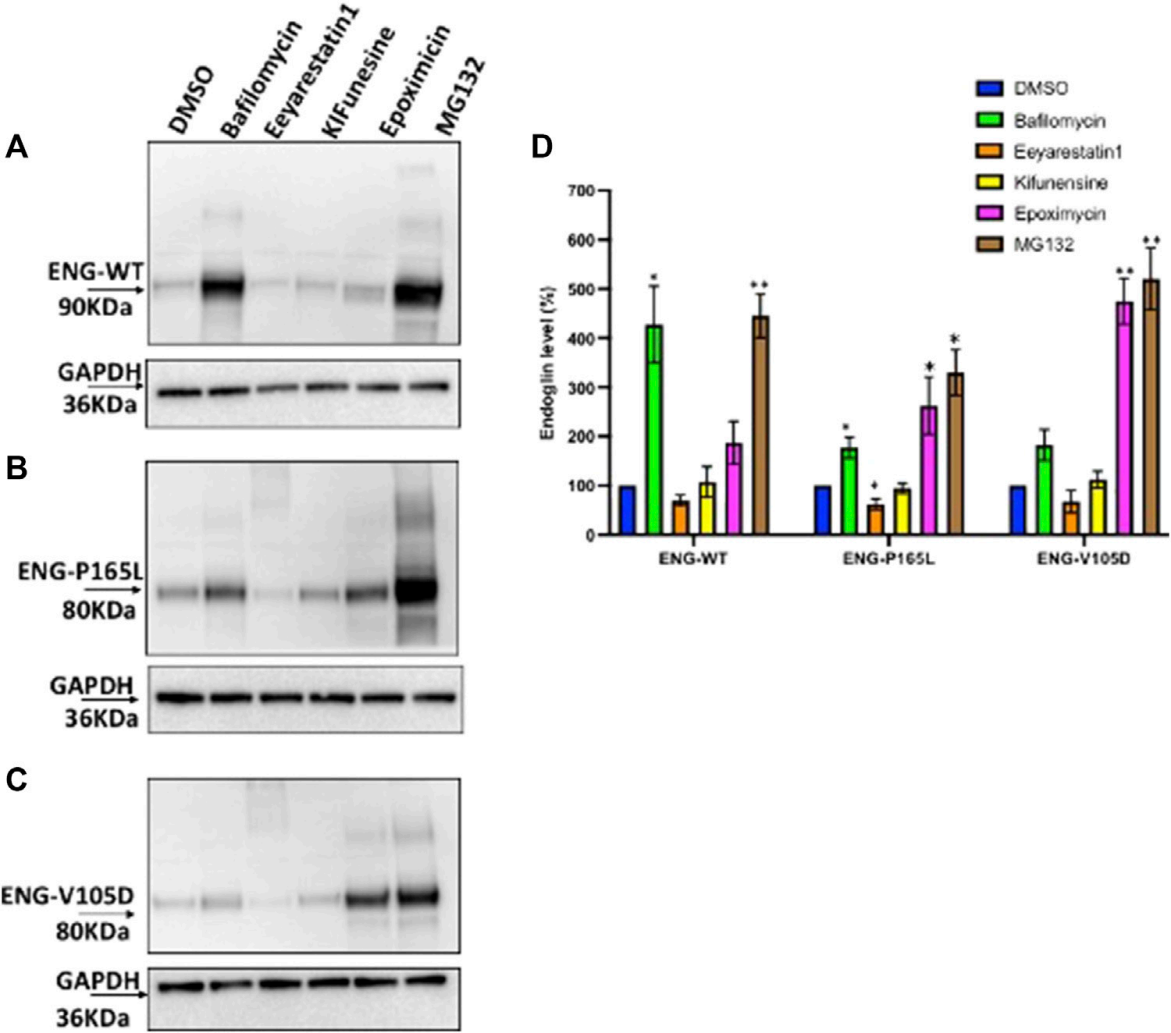
Degradation pathway of WT and mutant Endoglin P165L and V105D. **(A–C)** Stable HEK293 cell lines harboring the HA-tagged ENG-WT and mutant variants p. P165L and p. V105D were treated with Bafilomycin (200 nM) (lysosomal inhibitor), Eeyrestatin I (5 μM) and Kifunesine (50 nM) (ERAD inhibitors), MG132 (10 μM), and Epoximycin (100 nM) (proteasomal inhibitors). Total cell lysate was analyzed by immunoblotting against antibodies for HA- ENG and GAPDH. **(D)** Bar graphs representing mean densities of WT Endoglin and mutants normalized with GAPDH. Endoglin level was expressed in (%) relative to DMSO treated (control). Bars represent SEM from 4 different Experiment (*n* = 4). Statistical significance for each treatment relative to control was assessed using one sample t test, (*) *p* ≤ 0.05; (**); *p* ≤ 0.01; (***) *p* ≤ 0.001.

HEK293 cells stably transfected with Endoglin WT, P165L and V105D were treated individually with Bafilomycin, Eeyarestatin I, Epoximycin and MG132 for 24 h then harvested and cell lysates were used for western blotting analysis to examine their stability under these treatments. Immunoblots generated in Figures 5A–C have been analyzed by densitometric analysis and illustrated in bar graph (Figure 5D) depicting accumulation levels of Endoglin relative to DMSO treated cells (control). These results show significant accumulation of WT Endoglin (over 4 folds) compared to DMSO only treated cells, when incubated with the lysosomal inhibitor Bafilomycin (Figures 5A,D). On the other hand, variants P165L, and V105D have shown a slight accumulation (∼1.8 folds), when treated with Bafilomycine (Figures 5B–D). Treatment with the proteasomal inhibitors (MG132) have also resulted in significant accumulation of WT Endoglin (∼4.5 folds), however treatment with the other proteasomal inhibitor (Epoximycin) resulted in non-significant accumulation level (Figures 5A,D). On the other hand, unlike WT Endoglin, both Endoglin variant (P165L and V105D) have shown significant accumulation level when treated Epoximycin (2.5 and 4.5 folds), respectively (Figures 5B–D). Furthermore, treatment with MG132 have also resulted in significantly high level of accumulation for both Endoglin variants P165L and V105D (∼5.2, and 5.5 folds), respectively (Figures 5B–D). ERAD inhibition was achieved through the small molecules kifunensine (Kif) and Eeyarestatin I (EerI). EerI inhibits the deubiquitination mechanism associated with p97/VCP that facilitates the efficient recycling of ERAD substrates through the proteasomal pore (Stevenson et al., 2016). Treatment with Kif, a potent inhibitor of mannosidase I enzyme, has not affected the level of either WT Endoglin or the two mutant variants. On the other hand it has also been observed that EerI treatment has affected the mobility of both Endoglin variants on the gel, which is represented by a high molecular weight smearing pattern on the immunoblots (Figures 5B,C), which most probably correspond to polyubiquitinated form of mutant variants P165L and V105D. This may also explain the drop of Endoglin level at the normal molecular weight (80–90 KDa), represented by a faint band at this position (Figures 5B–D). This result suggests that mutant variants P165L and V105D are most likely degraded via the ERAD machinery.

### Significant Accumulation of P165L and V105D Endoglin Variants as a Result of HRD1 Deficiency

**FIGURE 6:**
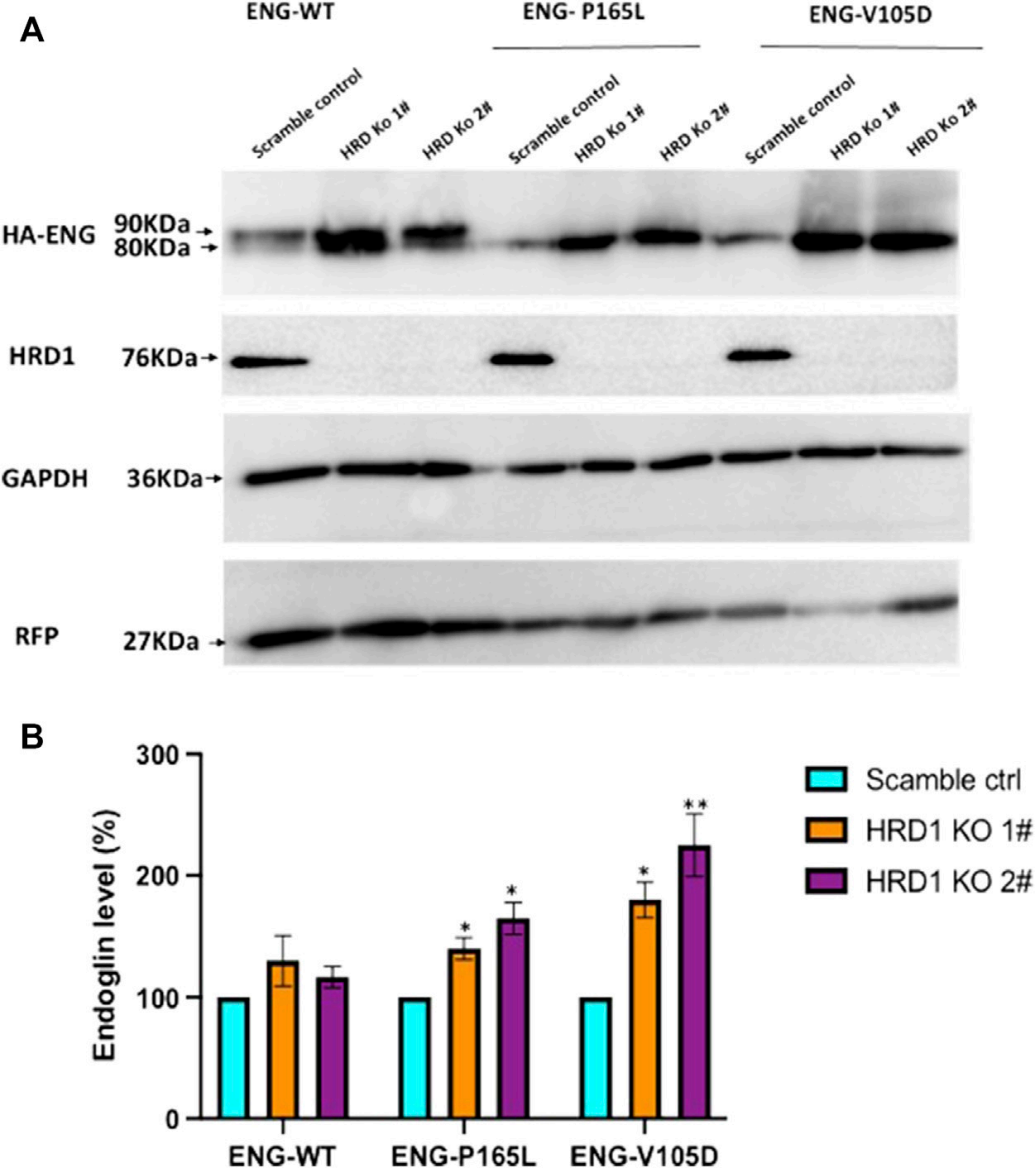
Endoglin WT and mutant variants accumulation level in HEK293^HRD1-KO^ cells. **(A)** Generated HRD1-Knockouts (1# and 2#) were transiently transfected with pcDNA3.0 plasmid vectors harboring WT Endoglin and mutant variants (P165L and V105D). Total cell lysate was analyzed by immunoblotting against HA-ENG, RFP, GAPDH, and HRD1 **(B)** Bar graphs representing mean densities of WT Endoglin and mutants normalized with GAPDH (loading control) and RFP (For transfection efficiency control). Endoglin level was expressed in (%) relative to (scramble control). Bars represent SEM from 4 different Experiment (*n* = 4). Statistical significance for Endoglin accumulation level in each cell line (relative to scramble control) was assessed using one sample t-test, (*) *p* ≤ 0.05; (**); *p* ≤ 0.01; (***) *p* ≤ 0.001.

Misfolded protein variants trapped in the ER lumen such as P165L and V105D are most likely recognized as ERAD substrates and subjected to retro-translocation through the HRD1/SEL1L translocon channel to be degraded in the cytosol through the proteasomal machinery. In order to investigate the role of such evolutionary translocon channel in the degradation pathway of Endoglin WT and mutant variants P165L and V105D, we have utilized CRISPR-Cas9 gene editing technology to create HEK293 cell lines deficient in HRD1 E3 Ubiquitin ligase. HRD1 knockout was validated using Sanger sequencing and immunoblotting against HRD1 (Supplementary Figure S4; Figure 6A), respectively. Clonal HEK293 ^HRD1−KO^ cells were individually transiently transfected with plasmid vectors harboring WT Endoglin and mutant variants P165L and V105D (Figure 6). For the purpose of transfection efficiency control, cells were co-transfected with RFP plasmid vector. Immunoblotting results have shown that Endoglin variants P165L and V105D have significant protein accumulation in the two HEK293 ^HRD1−KO^ cell lines (HRD1 KO 1# and HRD1 KO 2#) compared to that observed in HEK 293^Sc^ (scrambled control) (Figures 6A,B). These findings emphasize the essential role of HRD1 E3 ubiquitin ligase in the degradation pathway of misfolded Endoglin variants that further implicates the role of ERAD machinery in the pathology of HHT1. On the other hand, WT Endoglin has shown insignificant accumulation level in HEK293 ^HRD1 KO^ cells, which could indicate that proteasomal degradation of WT Endoglin does not process significantly through the HRD1/SEL1 translocon channel.

## Discussion

Secretory proteins enter the ER through a translocon pore in an unfolded state, where they may undergo several posttranslational modifications. These include N-glycosylation, proline isomerization and disulfide bond formation, assembly of multi-subunit complexes facilitated by molecular chaperones that guide protein folding, and various complex assembly processes (Cherepanova et al., 2016). Due to the stringent quality control mechanisms dedicated to protein folding, it was estimated that 12–15% of newly synthesized protein in the ER are eliminated co-translationally through the proteasomal system (Sun and Brodsky 2019). The ER quality control mechanism (ERQC) is a surveillance mechanism which ensures that, in order to proceed to the next compartment in the secretory pathway, all proteins fit a predetermined criteria that define the “normal”. The problem is exacerbated when proteins persistently fail to reach their native folding states due to disease-causing mutations (Chen et al., 2005). Some aberrant, but partially functional proteins can be prematurely selected for degradation by the ERQC mechanism, which can lead to their loss of function within cells (Shao and Hegde 2016). A classic example of this process is the degradation of certain mutant variants of CFTR (cystic fibrosis transmembrane conductance regulator) associated with cystic fibrosis disease (Cheng et al., 1990).

Components of the TGFβ receptor complex, including Endoglin, are examples of plasma membrane proteins that enter the secretory pathway and get scrutinized by the ERQC system, transported to the Golgi for additional specific glycosylation, and then trafficked to their functional location at the plasma membrane (Fisher et al., 2019). We have previously reported that defective trafficking can prevent membrane localization of Endoglin, TGFβ Type II receptor (TGFβ II), BMP type II receptor (BMPR II), and ALK1, (John et al., 2015). This leads to defective folding, retention in the ER, followed by possible ERAD targeting, and protein elimination (John et al., 2015; Meurer et al., 2019).

In this report, for the first time, we demonstrate that misfolded Endoglin variants trapped in the ER are more stable than WT Endoglin localized at the plasma membrane. Moreover, our results have shown that misfolded Endoglin variants V105D and P165L are predominantly degraded through the proteasomal pathway, whereas Endoglin WT is degraded through both the proteasomal and lysosomal pathways.

This result was intriguing since the proteasomal system is essential for intracellular protein degradation, but an extracellular role of this degradation machinery has rarely been reported (Banik et al., 2020; Sawada et al., 2002). As mentioned above, the protein folding process is error prone, therefore WT membrane proteins are frequently degraded intracellularly through the proteasomal pathway when they temporarily fail to reach their proper conformation (Shao and Hegde 2016). It is a process that has been adapted by the ERQC mechanism in order to maintain protein homeostasis in the ER. Nonetheless, our results also demonstrate that inhibition of the lysosomal pathway results in excessive accumulation of WT Endoglin, which is most likely attributed to the fully glycosylated mature receptor protein localized at the plasma membrane. This finding could open a new therapeutic target that might relieve the haploinsufficiency state via moderate inhibition of WT Endoglin lysosomal degradation. However, the process will require specific pharmacological agents which modulates WT Endoglin degradation without an overall inhibition of the lysosomal pathway.

Lysosomal degradation has also been implicated in the degradation process of another TFGβ receptor; BMPR2, associated with familial Pulmonary hypertension (HPAH). It is a genetic disease characterized by elevated pulmonary pressure due defective arterial formation (Gomez-Puerto et al., 2019). The study demonstrates that in addition to proinflammatory cytokines, BMPR2 heterozygosity can cause augmentation of the autophagic influx, which could be secondary to the role of BMPR2 signaling in autophagy regulation (Gomez-Puerto et al., 2019). We have demonstrated in a previous study that a number of disease-causing variants of BMPR2 are trapped in the ER (John et al., 2015). Therefore, defective trafficking followed by premature degradation of these variants through ERAD was proposed as the most likely mechanism underlying the disease’s loss of function phenotype. Thus far, no study has investigated the degradation pathway of these variants and therefore the molecular mechanism underlying the disease pathology remains to be elucidated.

The ERAD mechanism has become evidently implicated in the pathogenesis of numerous diseases including cystic fibrosis, emphysema, acromesomelic dysplasia-type Maroteaux, Robinow syndrome, and various neurodegenerative diseases such as Parkinson and Alzheimer (Chen et al., 2005; Hume et al., 2013; Kaneko et al., 2017). In a recent review, we proposed investigating the role of the ERQC mechanisms including ERAD, in the pathology of various diseases associated with the TGFβ component including HHT1 (Gariballa and Ali 2020). In this study, we have demonstrated that mutant variants trapped in the ER are prematurely degraded through the proteasomal pathway which directly implicates the ERAD mechanism in the degradation process. Variants P165L and V105D have shown a longer cellular half-life than the WT Endoglin, possibly triggering the UPR mechanism and a cascade of events that usually leads to the elimination of misfolded proteins (Christianson and Ye 2014; Wu and Rapoport 2018). In order to further investigate the implication of ERAD components in the degradation pathway of these mutant variants, we utilized CRISPR Cas9 editing technology in order to investigate a direct involvement of major ERAD components in the degradation pathway of Endoglin variants. Here, we have demonstrated that deficiency of HED1 E3 ubiquitin ligase in HEK293 cells causes accumulation of misfolded variants of Endoglin retained in the ER. These results indicate that HRD1/SEL1L retro-translocon channel plays a key role in the elimination of these misfolded variants. In mammalians, a number of E3 ubiquitin ligases have been reported to play a role in the ubiquitination of ERAD substrates including HRD1, gp78, RMA1/RNF5, TEB4, TRC8/RNF139, RNF170, RNF103, and RFP2/TRIM1, which are all possible candidates for ERAD substrate ubiquitination (Christianson and Ye 2014; Kadowaki et al., 2018). TRIM21/Ro52 E3 ubiquitin ligase was previously found to play a role in the proteasomal degradation of unfolded IgG1 (Takahata et al., 2008). Recently, it has also been shown to interact with soluble Endoglin (sEng); a circulating proteolytic product of the transmembrane receptor protein (Gallardo-Vara et al., 2019). However its role in the proteasomal degradation of mutant variants of Endoglin transmembrane receptor remains to be elucidated. Nevertheless, the purpose behind the availability of numerous E3 ligases dedicated to ERAD substrates ubiquitination is still unclear. However, variability of ERAD substrates in terms of their target destination (cytosolic, plasma membrane, etc.) and mutation localization could be one of the reason for the dedication of this huge number of E3 ubiquitin ligases embedded in the ER membrane. Nonetheless, HRD1 has been one of the most studied and best characterized E3 ligase and has recently been identified as a core component of ERAD substrates ubiquitination (Carvalho et al., 2010; Baldridge and Rapoport 2016; Schoebel et al., 2017; Kadowaki et al., 2018; Wu et al., 2020; Taguchi et al., 2021). Loss of HRD1 has been reported to be directly linked to accumulation of amyloid β (Aβ) implicated in Alzheimer Disease (Kadowaki and Nishitoh 2013). Accumulation of Aβ triggers the UPR mechanism which resolves the situation by inducing apoptosis, leading to neurodegeneration (Calabrò et al., 2021). HRD1 has also been shown to accelerate the degradation of cytotoxic aggregates of polyglutamine (polyQ) involved in development of Huntington disease (Yang et al., 2007). Interestingly, in this study, we see a reversed scenario where ERQC components such as HRD1 play a role in a premature degradation mechanism leading to a pathological condition characterized by loss of function traits. Premature mutant protein degradation through the proteasomal pathway has also been the underlying cause for certain classes of Cystic fibrosis (CF) (Meacham et al., 2001). Extensive work has been done in order to investigate CFTR biogenesis including protein folding and trafficking (Glozman et al., 2009; Balch et al., 2011). Identification of key players in the CF pathology has resulted in various classes of CF modulators, achieving remarkable progress in personalized treatment for CF patients (Lopes-Pacheco 2016). Therefore, we predict that a more in-depth investigation into the molecular mechanisms that orchestrate premature elimination of Endoglin variants is likely to result a wider range of therapeutic targets.

In conclusion, this study has demonstrated that ER retained Endoglin variants P165L and V105D show high stability in the ER lumen compared to WT Endoglin, however they are not likely to form permanent insoluble aggregates. Furthermore, we have shown that these mutant variants degrade through the proteasomal pathway and very likely through the ERAD mechanism, while WT Endoglin seem to degrade through both lysosomal and proteasomal pathways. Although our results have shed a light on the degradation mechanism of WT Endoglin and two disease causing variants, we still need to extend our studies to a wider range of cell lines including HHT1 patients’ Endothelial cells. Furthermore, the molecular mechanisms of Endoglin biogenesis including chaperone assisted folding, trafficking and protein fidelity checkpoints must be elucidated in order to design specific modulators for various types Endoglin variants.

## Data Availability

The data upon which conclusions have been made in this article are included in the article/Supplementary Material section. Further inquiries can be directed to the corresponding author.
