# Supplementary figures and images for: Endoglin Wild Type and Variants Associated With Hereditary Hemorrhagic Telangiectasia Type 1 Undergo Distinct Cellular Degradation Pathways
Source: Front Mol Biosci. 2022 Feb 25;9:828199. doi: 10.3389/fmolb.2022.828199 (PMC8916587; doi:10.3389/fmolb.2022.828199)

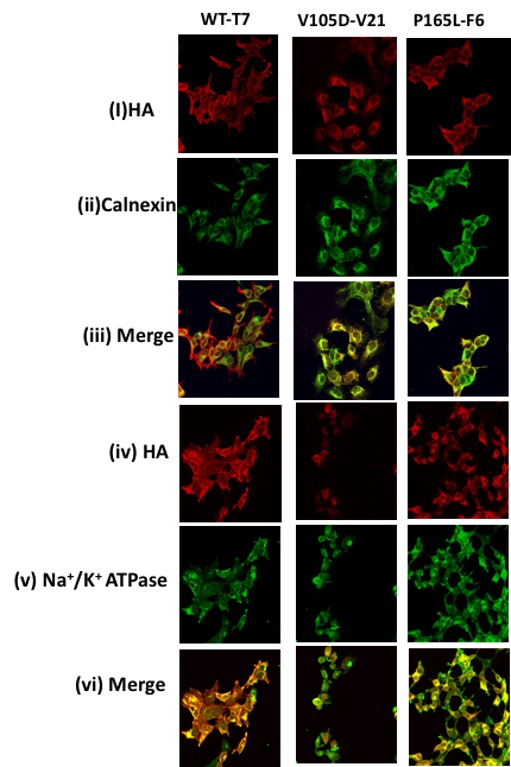

Supplement: Supplementary file 1 [file Image3.JPEG]

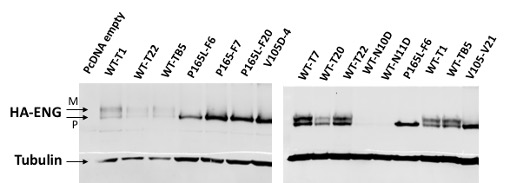

Supplement: Supplementary file 2 [file Image1.JPEG]

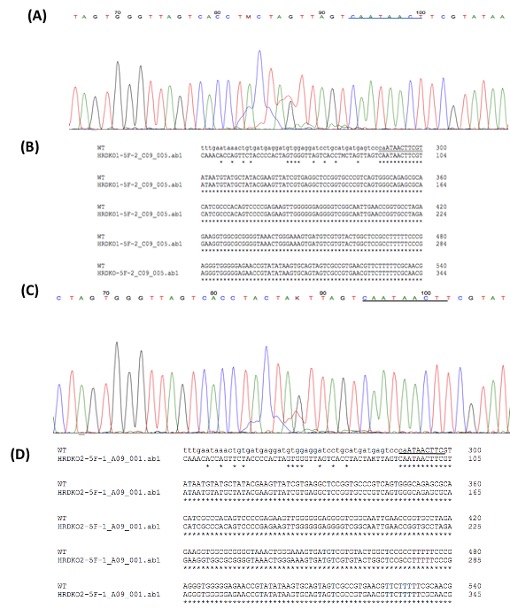

Supplement: Supplementary file 3 [file Image4.JPEG]

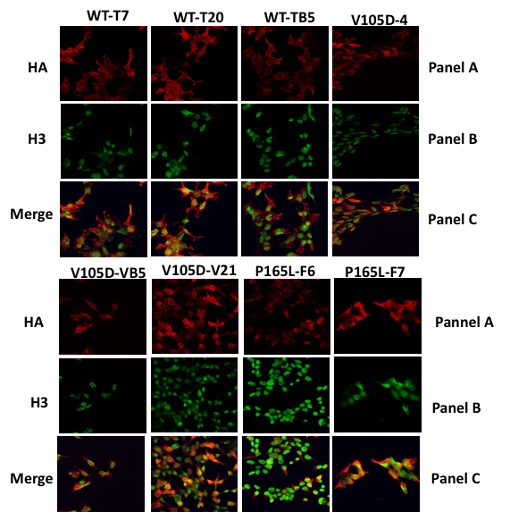

Supplement: Supplementary file 4 [file Image2.JPEG]
