# Supplementary material for: Endoglin Wild Type and Variants Associated With Hereditary Hemorrhagic Telangiectasia Type 1 Undergo Distinct Cellular Degradation Pathways
Source: Front Mol Biosci. 2022 Feb 25;9:828199. doi: 10.3389/fmolb.2022.828199 (PMC8916587; doi:10.3389/fmolb.2022.828199)
Supplement: Supplementary file 5 [file DataSheet1.docx]

**Supplementary Figures Legend**

**Supplementary Figure S1: (A)** Immunoblots show stable expression of ENG WT and mutant variants P165L and V105D in various HEK293 mono-clones (ENG-WT-T1, T7, T20, T22, TB5) , (ENG-P165-F6, P165L-F7, P165L-F20) and (ENG-V105D-4, V105D-V21). WT Endoglin is represented in the blot by a fully glycosylated mature protein band (M) at ~90KDa and a precursor band (P) at ~80KDa, whereas the mutant variants appear as a single immature band at 80KDa

**Supplementary Figure S2**: Immunofluorescence analysis of Endoglin stable monoclones. HEK 293 cells stably expressing the wild type (Clones WT-T7, WT-T20, WT-TB5), V105D mutant (clones V105D-4, V105D-V21) and P165L (Clones P165L-F6, P165L-F7) showing the localization pattern of HA-tagged ENG (Red, top panel). To demarcate the cells, anti-Histone H3 antibodies targeting the nuclei (green) was used. Bottom panel shows the merged image from the two channels.

**Supplementary Figure S3**. Immunofluorescence images show immunostaining against Endoglin HA-tag (Red) in HEK293 monoclones stably transfected with WT Endoglin and the two mutant variants P165L and V105D. Calnexin (Panel (ii), green)and Na^+^/K^+^ antibody (Panel (v), green) were used as endogenous markers for endoplasmic reticulum (ER) and plasma membrane (PM) respectively. The wild type shows co-localization with the plasma membrane marker while the mutants show co-localization with the ER-marker.

**Supplementary Figure S**4**. HRD1 Knock-out validation in single cell clones of HEK293 cells.** Panels **(A)** and **(C)** represent sequencing chromatogram of amplified target sequence in KO1 and KO2 HRD1 KO clones, respectively. Panels **(B)** and **(D)** represent the alignment of each of the knockouts’ PCR product sequence, generated by Sanger sequencing, with WT sequence using Clustal Omega software. The starting point of alignment between HRD1 wild type sequence and Knockout clones is underscored in both sequencing chromatogram and Clustal Omega sequence alignment.
